# Comparative diagnostic performance of metagenomic next-generation sequencing and conventional microbial culture in spinal infections: a systematic review and meta-analysis

**DOI:** 10.3389/fcimb.2026.1689254

**Published:** 2026-03-13

**Authors:** Binyue Zhang, Limei Wang, Jing Wang, Dongxu Qi, Na Zhang

**Affiliations:** 1 Clinical Laboratory Department, Jilin Province FAW General Hospital, Changchun, China; 2 Department of Pharmacy, Jilin Province FAW General Hospital, Changchun, China; 3 Preventive Medicine Department, Jilin Province FAW General Hospital, Changchun, China; 4 Department of Geriatrics, Jilin Province FAW General Hospital, Changchun, China; 5 Electrodiagnosis Department, Jilin Province FAW General Hospital, Changchun, China

**Keywords:** conventional microbial culture, meta-analysis, metagenomic next-generation sequencing, spinal infections, systematic review

## Abstract

**Background:**

Spinal infections are relatively uncommon but clinically serious conditions that require timely and accurate diagnosis to prevent severe complications. Traditional microbial culture methods remain the gold standard but suffer from low sensitivity and prolonged turnaround times. Metagenomic next-generation sequencing (mNGS) has emerged as a promising diagnostic tool offering broad-spectrum pathogen detection. However, its diagnostic performance in spinal infections remains unclear.

**Objective:**

To systematically evaluate and compare the diagnostic accuracy of mNGS and conventional microbial culture in detecting pathogens in spinal infections.

**Methods:**

This systematic review and meta-analysis adhered to the 2020 PRISMA guidelines and was registered in PROSPERO. A comprehensive literature search of PubMed, Cochrane Library, Web of Science, and Scopus was performed up to July 2025. Studies involving suspected spinal infection patients tested by both conventional microbiological methods and metagenomic next-generation sequencing (mNGS) were included. Data extraction and quality assessment were independently conducted by two reviewers using standardized tools. Meta-analyses were performed to pool diagnostic accuracy metrics, and publication bias was assessed.

**Results:**

A total of 14 studies involving 1,353 patients were included after screening 4,132 records. All studies originated from China, with sample sizes ranging from 17 to 301. Quality assessment showed generally high methodological rigor with low risk of bias. Conventional meta-analysis demonstrated that mNGS had significantly better positive agreement (OR = 0.46, p < 0.00001), higher sensitivity (OR = 0.45, p < 0.00001), and superior negative predictive value (OR = 0.36, p < 0.00001) compared to traditional methods, while specificity and positive predictive value were comparable. Diagnostic meta-analysis revealed pooled sensitivity and specificity of 0.86 and 0.90, respectively, with an AUC of 0.90, indicating high diagnostic accuracy. Fagan nomogram analysis showed that with a 50% pre-test probability, positive and negative mNGS results corresponded to post-test probabilities of 89% and 13%, respectively. No significant publication bias was detected.

**Conclusions:**

mNGS exhibits superior sensitivity and overall diagnostic accuracy compared to traditional microbial culture in spinal infections, supporting its use as a valuable complementary diagnostic tool. Further prospective, multicenter studies are warranted to validate these findings and promote standardized clinical implementation.

**Systematic Review Registration:**

PROSPERO, identifier CRD420251114975.

## 1 Introduction

Spinal infections represent a relatively rare but clinically serious group of infectious diseases that typically involve the vertebral body, intervertebral discs, and surrounding structures (Tay et al., 2002; An and Seldomridge, 2006; Mazzie et al., 2014). Patients may present with symptoms such as fever, back pain, and neurological deficits (Diehn, 2012; Yao et al., 2018; Tan et al., 2020). Without timely diagnosis and intervention, spinal infections may lead to structural destruction, abscess formation, or even paralysis. Due to their atypical early manifestations and the limited specificity of imaging and laboratory indicators, early diagnosis of spinal infections remains a clinical challenge (Spock et al., 2006; Caputo et al., 2013; Babic and Simpfendorfer, 2017).

Conventional microbiological diagnostic methods—such as culture, staining, and polymerase chain reaction (PCR)—still dominate clinical practice but suffer from limitations such as low sensitivity, long turnaround times, and markedly reduced positivity rates after antibiotic treatment (Khanna and Sabharwal, 2019; Lai et al., 2019). These methods also struggle to identify hard-to-culture, atypical, or polymicrobial pathogens. Recently, metagenomic next-generation sequencing (mNGS), a broad-range, target-independent diagnostic approach, has demonstrated excellent pathogen detection capabilities in central nervous system infections, pulmonary infections, and bloodstream infections (Guo et al., 2022; Xie et al., 2023; Yang et al., 2023). The term ‘Next-Generation Sequencing’ (NGS) refers to the current high-throughput sequencing technologies that are widely used in clinical practice for pathogen detection and microbiological diagnosis. While the term was originally associated with future advancements in genomic research, in this study, we refer to it as the established and applied technology used in diagnosing spinal infections. Its role as a supplementary tool in the diagnosis of challenging infections is gaining increasing attention.

However, there is still no consensus regarding the actual diagnostic performance of mNGS in spinal infections. While its potential advantages include high sensitivity, rapid turnaround, and broad-spectrum pathogen detection, its clinical application remains controversial due to variability in workflow, interpretation standards, and study designs (Li et al., 2025). To date, there is a lack of systematic evidence quantifying its sensitivity, specificity, and overall diagnostic value, and no studies have comprehensively validated its clinical applicability using robust statistical methods.

To address this evidence gap, we conducted a systematic review and meta-analysis. By combining findings from conventional meta-analyses and diagnostic accuracy analyses, our objective was to compare the diagnostic performance, including concordance, sensitivity, specificity, and predictive value, of mNGS with conventional microbial culture in spinal infections. This work is intended to provide evidence-based support for clinical decision-making and to promote the establishment of standardized diagnostic protocols in infectious disease practice.

## 2 Methods

### 2.1 Search strategy

This study was conducted in accordance with the 2020 PRISMA guidelines (Liberati et al., 2009; Hutton et al., 2015; Shamseer et al., 2015). The review protocol was registered in PROSPERO (registration number: CRD420251114975). A systematic search of PubMed, Cochrane Library, Web of Science, and Scopus was conducted, with the last search updated in July 2025. Detailed search strategies are provided in the **Supplementary Material**.

To minimize the risk of missing eligible studies, we also manually screened the reference lists of previously published meta-analyses to ensure inclusion of all relevant high-quality studies, thereby minimizing selection bias and enhancing comprehensiveness and reliability.

### 2.2 Inclusion and exclusion criteria

Studies were selected based on the PICOS framework (Population, Intervention, Comparator, Outcome, Study design):

- Population: Suspected spinal infection patients meeting the diagnostic criteria of the Infectious Diseases Society of America (IDSA) guidelines for adult pyogenic vertebral osteomyelitis; tuberculosis-related spinal infections were excluded. This exclusion was based on the significant differences in the pathology, clinical presentation, and treatment strategies of tuberculosis-related spinal infections compared to other spinal infections, which could affect the diagnostic methods’ comparability. Studies with incomplete data, such as missing true positive, true negative, false positive, and false negative counts, were excluded to ensure the reliability and completeness of the data for analysis (Khanna and Sabharwal, 2019; Li et al., 2023).
- Intervention: All included cases underwent both traditional microbiological testing and mNGS.
- Comparator: Conventional microbiological testing served as the reference to evaluate the diagnostic accuracy of mNGS.
- Outcomes:

Conventional meta-analysis focused on:

*Positive agreement*: Consistency between mNGS and the reference standard (histopathology, microbiological culture, or composite clinical diagnosis) in detecting true positives. Positive agreement was calculated as the proportion of true positives detected by both mNGS and the reference standard. This metric helps assess how often both methods agree on the presence of infection, providing additional insight into the concordance between diagnostic methods (Calica et al., 2025; Flores et al., 2025).

*Sensitivity*: Ability of the method to correctly identify infected individuals, critical for early screening and preventing neurological complications (Olin and Bartges, 2015; Zeng et al., 2024).

*Specificity*: Ability to correctly identify non-infected individuals, important for avoiding unnecessary antibiotic use and distinguishing infection from inflammation or neoplasms (Gregoire et al., 2022).

*Positive Predictive Value (PPV)* and *Negative Predictive Value (NPV)*: Reflect the clinical interpretability of test results, influenced by disease prevalence and relevant to clinical decision-making (Bouthry et al., 2021).

Diagnostic meta-analysis evaluated true positive (TP), false positive (FP), false negative (FN), and true negative (TN) rates to calculate diagnostic accuracy metrics (Qu et al., 2022; Tufano et al., 2023).

• Study Design: Only cohort studies with control groups and randomized controlled trials (RCTs) were included. Case reports, reviews, abstracts, commentaries, and study protocols were excluded.

### 2.3 Data extraction and risk of bias assessment

Two independent investigators (LW and NZ) performed data extraction using a standardized form. The extracted data included the first author’s name, year of publication, patient demographic characteristics (such as gender), total sample size, country or region where the study was conducted, study design, specimen collection method, and type of specimen analyzed. Disagreements were resolved through discussion with a third reviewer (BYZ).

For conventional meta-analysis, the Newcastle–Ottawa Scale (NOS) was used to evaluate the quality of retrospective studies (Lo et al., 2014). The scale assesses study selection, comparability, and outcome, with a maximum score of 9. Higher scores indicate higher methodological quality. For diagnostic meta-analysis, the QUADAS tool was used, covering 14 methodological criteria including population representativeness, sample selection, reference standard clarity, blinding, gold standard bias, follow-up adequacy, and consistency of test procedures (Oliveira et al., 2011; Yang et al., 2021; Lee et al., 2022).

### 2.4 Statistical analysis

Conventional meta-analysis was conducted using Review Manager 5.4. Categorical variables were pooled as odds ratios (OR), continuous variables as mean differences (MD) (Leite et al., 2020). The random-effects model was used to account for potential heterogeneity between studies. Publication bias was assessed using funnel plots, and forest plots were used to illustrate individual study results and pooled estimates. Heterogeneity was evaluated using the Chi-squared test and I² statistics. I² values greater than 50% were considered indicative of substantial heterogeneity, and sensitivity analyses were performed to explore possible sources of heterogeneity.

Diagnostic meta-analysis was performed using STATA 18.0 (Bagos, 2015; Shim et al., 2017). Pooled sensitivity, specificity, positive likelihood ratio (PLR), negative likelihood ratio (NLR), and diagnostic odds ratio (DOR) were calculated. The summary receiver operating characteristic (SROC) curve and the area under the curve (AUC) were used to evaluate the overall diagnostic performance. Publication bias was assessed using Deek’s funnel plot. All statistical tests were two-sided, and a p-value of <0.05 was considered statistically significant.

## 3 Results

### 3.1 Literature search and study selection

**Figure 1:**
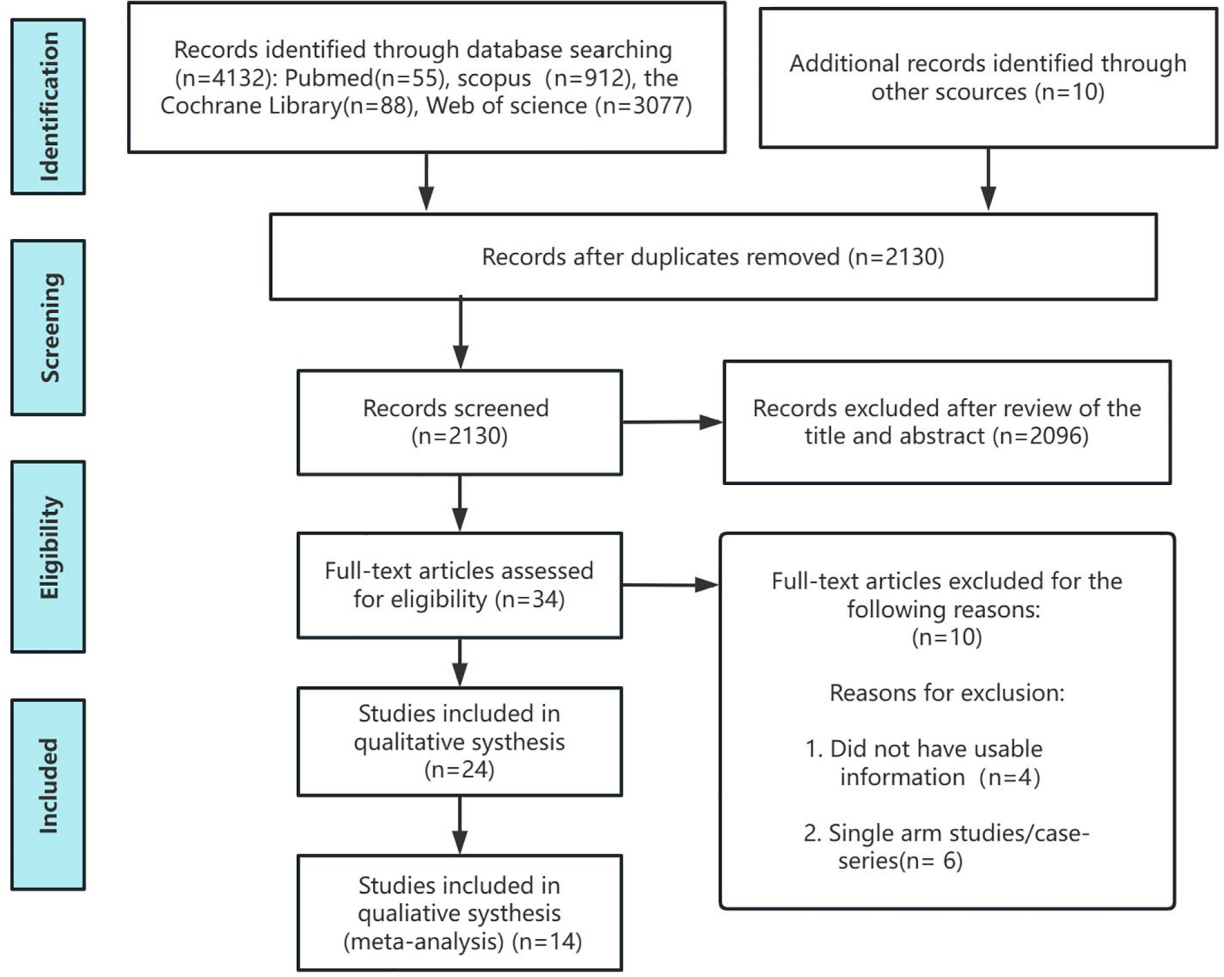
Flow chart of the study selection process for the meta-analysis.

**Figure 1** presents the PRISMA flowchart. A total of 4,132 records were retrieved. After removing 2,002 duplicates, 2,130 unique articles were screened. Following full-text review and application of inclusion criteria, several studies were excluded. For example, Li 2023 (Li et al., 2023) Zhang 2024 (Zhang et al., 2024), and Li 2022 (Li et al., 2022) focused on spinal tuberculosis and were excluded due to incompatibility with our study population. Wan 2022 (Wan et al., 2022) and Li 2022 (Li et al., 2022) were excluded for targeting spinal cord injury with sepsis and isolated Klebsiella pneumoniae infections, respectively. Du 2023 (Du et al., 2023) was excluded as it was a single case report (**Supplementary Table 1**). Ultimately, 14 studies involving 1,353 subjects were included in the meta-analysis.

### 3.2 Study characteristics

**Table 1** summarizes the characteristics of the 14 included studies (Ma et al., 2022; Xu et al., 2022; Zhang et al., 2022; Cheng et al., 2023; Huang et al., 2023; Lin et al., 2023; Wang et al., 2023; Wang et al., 2023; Zhang et al., 2023; Chen et al., 2024; Lv et al., 2024; Qi et al., 2024; Li et al., 2025; Yin et al., 2025). Publication years ranged from 2022 to 2025, and sample sizes varied from 17 to 301 patients. Most specimens were obtained intraoperatively or via image-guided puncture (CT, X-ray, or ultrasound). Notably, all studies originated from China.

**Table 1:** Characteristics of the studies that were included.

Author year	Country	Patients	Sex (Male/Female)	Study design	Collect samples	Sample type		Paired testing	Reference standard	Turnaround time
						mNGS	Conventional culture			
Zhang 2022	china	38	29/9	Retrospective	Surgical intervention (percutaneous transforaminal endoscopic debridement and drainage)	Tissue and pus samples	Blood and pus samples	Partially paired (mNGS: 56 tissue/pus samples; culture: 32 blood + 37 pus samples; not one-to-one matched)	Histopathology (gold standard)	NR (turnaround time not reported); samples sent for pathogen testing within 2 h of collection
Cheng 2023	china	78	52/26	Retrospective	Obtain samples from the lesion site	Tissue and pus samples	NR	Yes (lesion samples divided for culture; all cases underwent histopathology and mNGS)	Histopathology (reference standard)	mNGS ≤48 h; culture incubation up to 7 d
Wang 2023	china	25	NR	Retrospective	Via CT-guided percutaneous biopsy or debridement surgery	Specimen	Specimen	Partially paired (specimens split for culture/mNGS/pathology; culture missing in 2 cases)	Histopathology (used as reference standard)	mNGS 2.16 ± 0.69 d; culture 4.74 ± 1.71 d
Zhang 2023	china	158	81/77	Retrospective	Intraoperative acquisition of spinal lesion samples	Purulent soft tissue	Purulent soft tissue	Partially paired (mNGS + culture performed for 92 surgical specimens; additional cases without mNGS included)	Pathology-based diagnosis (pathological examination used as gold standard/inclusion criterion)	NR
Xu 2022	china	108	55/53	Retrospective	Surgical biopsy	Tissue specimen	Tissue specimen	Yes (specimens from each patient tested via mNGS and conventional microbiological tests)	Final clinical diagnosis (clinical golden standard; composite adjudication)	mNGS ~30 h; culture NR
Ma 2022	china	30	16/14	Retrospective	Surgical or CT-guided percutaneous biopsy	Specimen	Specimen	Yes (samples collected for mNGS, culture, and histopathology; surgical or CT-guided biopsy)	Histopathology (reference standard)	NR
Chen 2024	china	108	46/62	Retrospective	C-arm fluoroscopy-guided puncture or surgery	Pus, secretion samples, and tissue specimens	Pus, secretion samples, and tissue specimens	Partially paired (lesion tissue for mNGS/culture/pathology; 7 samples mNGS-only due to limited specimen)	Final clinical diagnosis (gold standard)	mNGS 1.54 ± 0.75 d; culture 3.09 ± 1.16 d
Lin 2023	china	39	19/20	Retrospective	Biopsy performed via percutaneous endoscopy or open surgery	Biopsy specimen	Biopsy specimen	Yes (biopsy specimens tested by both mNGS and culture; specimen split for culture/mNGS/pathology)	Pathologic test/histopathology (reference standard)	mNGS ≤24 h; culture confirmation within 72 h
Wang 2024	china	114	60/54	Retrospective	CT-guided percutaneous biopsy	Biopsy specimen	Blood and/or tissue	Yes (specimens split: tissue/blood for mNGS; remaining samples for culture/smear/pathology)	Final clinical diagnosis (also compared against CMT: culture and/or smear)	mNGS 29–53 h (mean 40.67 h); culture 90.88 ± 8.33 h
Yin 2025	china	120	80/30	Retrospective	CT-guided biopsy, C-arm fluoroscopy-guided biopsy, ultrasound-guided biopsy, or open surgery	Blood, tissue, or pus specimens	Blood, tissue, or pus specimens	Yes, pairwise comparisons	Pathology-based final diagnosis	mNGS ≤48 h; conventional tests 1–12 d
Li 2025	china	301	186/115	Retrospective	C-arm fluoroscopy- or CT-guided puncture	Tissue sample	Tissue sample	Yes (culture + mNGS performed for enrolled cases)	Composite clinical diagnosis (multidisciplinary final diagnosis)	mNGS 24–48 h; culture 2–7 d
Qi 2024	china	17	8/9	Retrospective	Invasive surgery	Purulent tissue and pus specimens	Purulent tissue and pus specimens	Partially paired (mNGS on intraoperative tissue/pus; conventional cultures mainly blood culture; tissue/pus also sent for culture in routine workup)	Pathology-confirmed infection (pathological examination used for final enrollment/diagnosis)	mNGS median 1.0 day; culture median ~5.88 days
Huang 2023	china	141	86/55	Retrospective	Obtained under fluoroscopy or CT guidance	Biopsy specimen	Biopsy specimen	Yes (PNB biopsy specimens underwent culture, histopathology, and mNGS)	Etiological + histopathological results (reference standard)	NR
Lv 2024	china	76	47/29	Retrospective	Percutaneous biopsy guided by color Doppler ultrasound puncture, endoscopy, or C-arm fluoroscopy	Biopsy specimen	Biopsy specimen	Yes (mNGS, culture, and histopathology completed simultaneously)	Composite clinical diagnosis (treatment response + pathology + imaging + labs)	mNGS mean 1.65 d; culture mean 3.07 d

### 3.3 Study quality and risk of bias

In the conventional meta-analysis, all retrospective cohort studies were evaluated using the Newcastle–Ottawa Scale (NOS), with scores ≥6, indicating high overall quality. Most studies scored ≥7 in the “Selection” and “Outcome” domains, reflecting reasonable design and comprehensive reporting. A few studies scored lower in the “Comparability” domain due to inadequate control of confounding variables. Overall, the methodological quality was acceptable (**Supplementary Table 1**).

**Figure 2:**
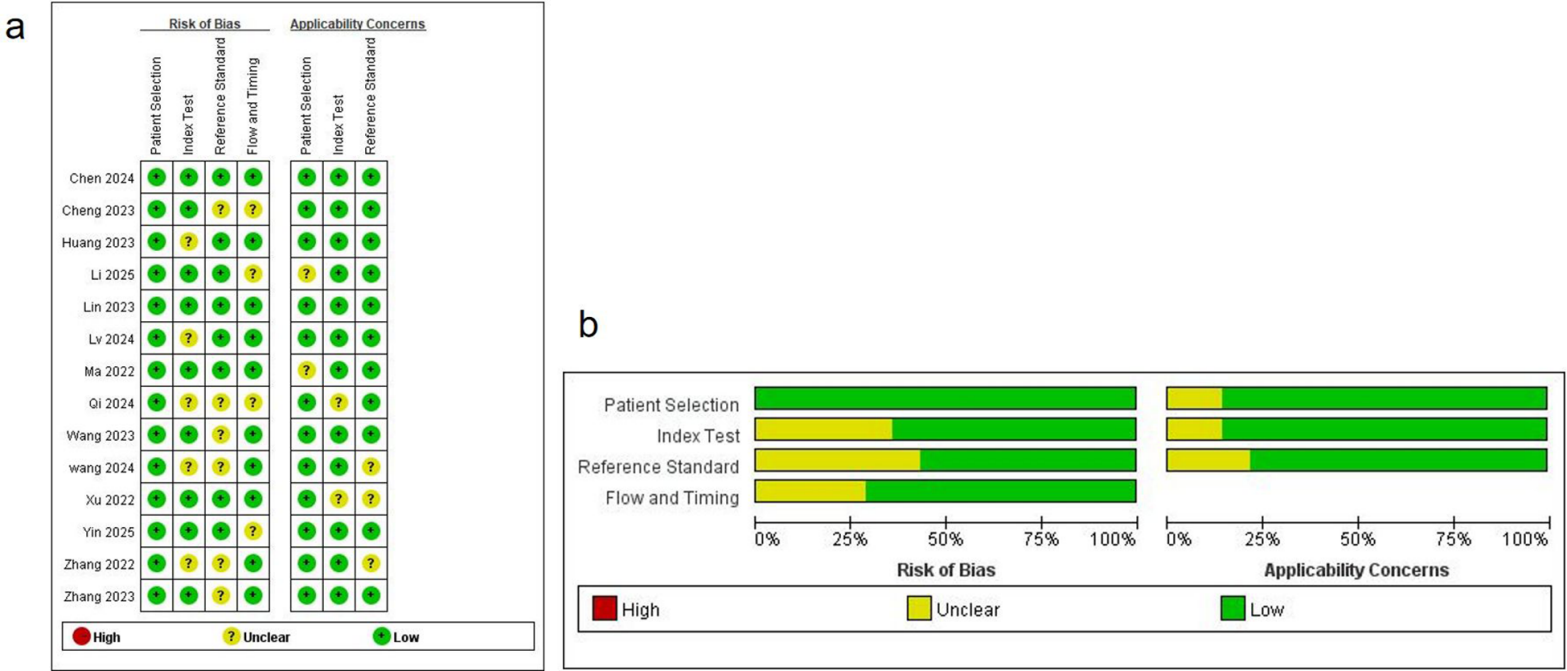
**(a)** Risk of bias and applicability concerns summary; **(b)** Risk of bias and applicability concerns graph.

In the diagnostic meta-analysis, study quality was assessed using the QUADAS-2 tool. As shown in **Figure 2**, most studies had low risk of bias in “Patient Selection,” “Reference Standard,” and “Flow and Timing.” However, six studies had an unclear risk of bias in the “Reference Standard” domain. This could be attributed to several factors: 1. Lack of clarity in reporting: Some studies did not clearly specify the reference standard or whether the interpretation of diagnostic tests was blinded to the reference results.2. Variability in reference standards: Different reference standards (e.g., histopathology, microbiological culture, clinical criteria) were used in some studies, and it was not always clear how these were integrated or compared. 3. Absence of a universally accepted reference standard: Since there is no single gold standard for diagnosing spinal infections, the variability in diagnostic criteria could lead to uncertainty regarding the reference standard. Six studies lacked sufficient information on whether index tests were interpreted without knowledge of reference results, and were thus rated as “unclear” in the “Index Test” domain. Nevertheless, applicability concerns were minimal, indicating a solid methodological foundation.

### 3.4 Conventional meta-analysis results

#### 3.4.1 Positive agreement

**Figure 3:**
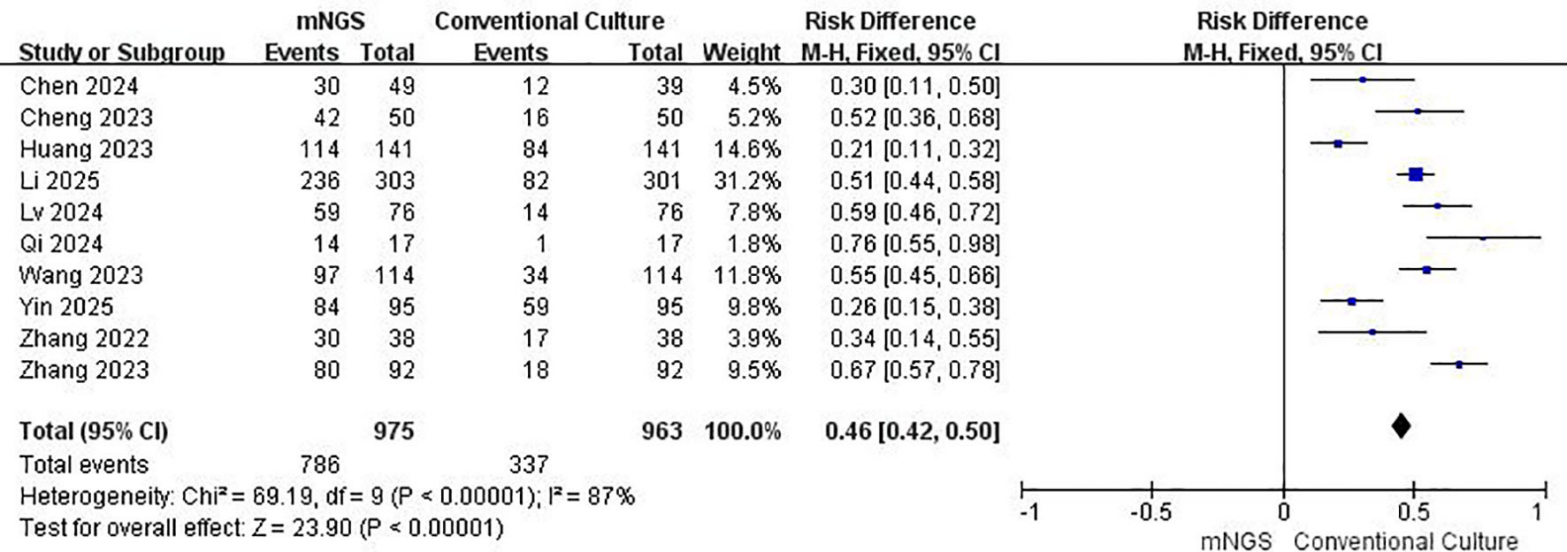
Forest plot for Positive Agreement, showing consistency between mNGS and the reference standard.

The funnel plot showed a symmetrical distribution (**Supplementary Figure 1a**), suggesting low publication bias. However, some studies fell outside the 95% confidence interval, indicating significant heterogeneity (I² = 87%). This could be due to differences in diagnostic standards or gold standards used across regions. As shown in **Figure 3**, pooled data from 10 studies revealed that mNGS had significantly better positive agreement than traditional methods (OR = 0.46, 95% CI: 0.42–0.50, p < 0.00001).

#### 3.4.2 Sensitivity

**Figure 4:**
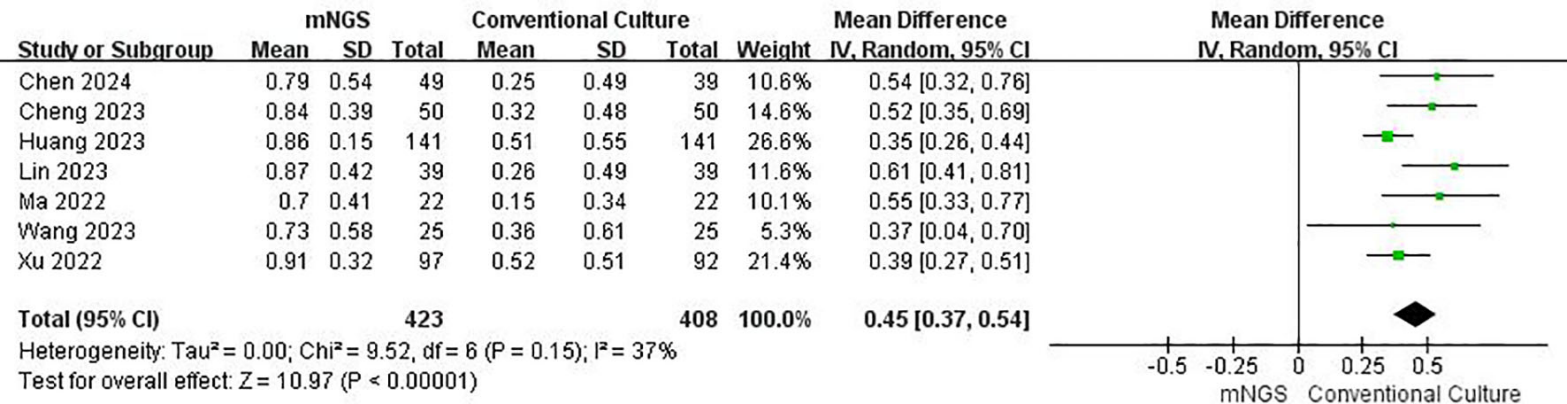
Forest plot for Sensitivity, illustrating the ability of mNGS to correctly identify infected individuals.

The funnel plot indicated symmetrical distribution (**Supplementary Figure 1b**) with low heterogeneity (I² = 37%). Based on seven studies (**Figure 4**), mNGS demonstrated significantly higher sensitivity than traditional microbiological methods (OR = 0.45, 95% CI: 0.37–0.54, p < 0.00001).

#### 3.4.3 Specificity

**Figure 5:**
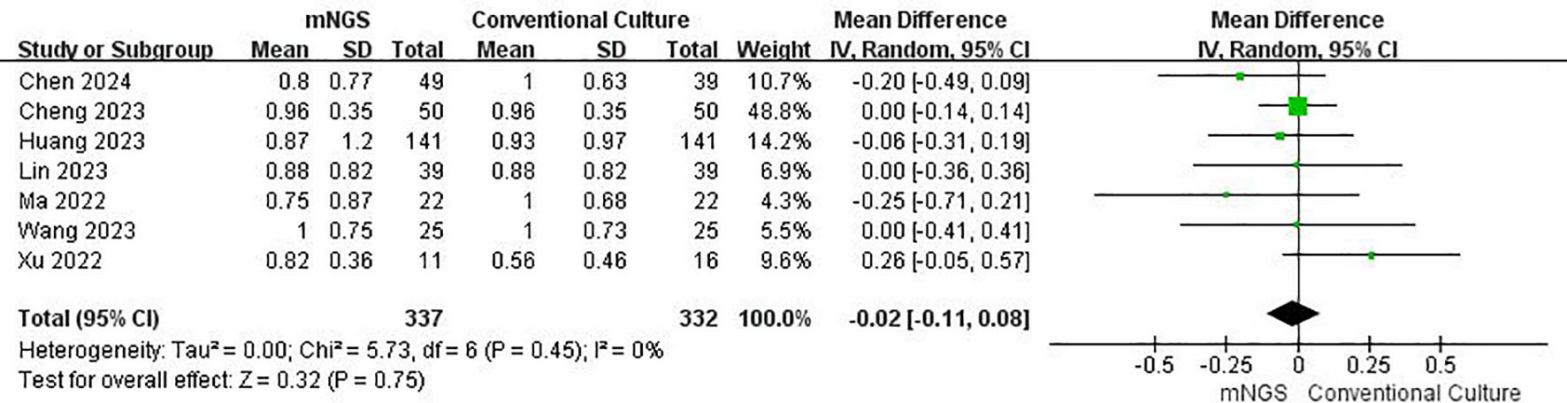
Forest plot for Specificity, demonstrating mNGS’s ability to correctly identify non-infected individuals.

The funnel plot for specificity showed no obvious asymmetry or publication bias (**Supplementary Figure 1c**). Forest plot analysis (**Figure 5**) revealed no significant difference between mNGS and traditional methods in specificity (OR = –0.02, 95% CI: –0.11–0.08, p = 0.75).

#### 3.4.4 Positive predictive value

**Figure 6:**
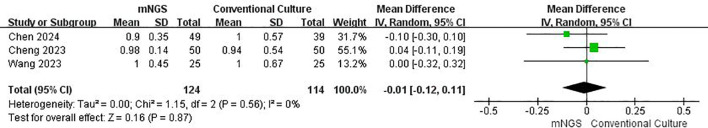
Forest plot for Positive Predictive Value (PPV), showing the clinical interpretability of mNGS results.

PPV funnel plot also showed symmetry (**Supplementary Figure 1d**). The pooled analysis from three studies (**Figure 6**) found no significant difference in PPV between mNGS and traditional methods (OR = –0.01, 95% CI: –0.12–0.11, p = 0.87).

#### 3.4.5 Negative predictive value

**Figure 7:**
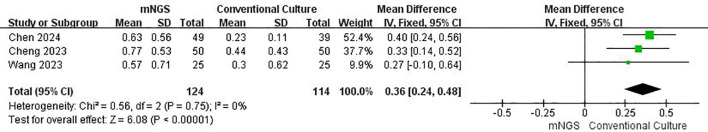
Forest plot for Negative Predictive Value (NPV), reflecting the test’s ability to rule out infections.

NPV analysis (**Supplementary Figure 1e**) indicated symmetrical data distribution and very low heterogeneity. Pooled results from three studies (**Figure 7**) showed that mNGS was significantly superior to traditional methods in NPV (OR = 0.36, 95% CI: 0.24–0.48, p < 0.00001).

### 3.5 Diagnostic meta-analysis results

#### 3.5.1 Pooled diagnostic performance

**Figure 8:**
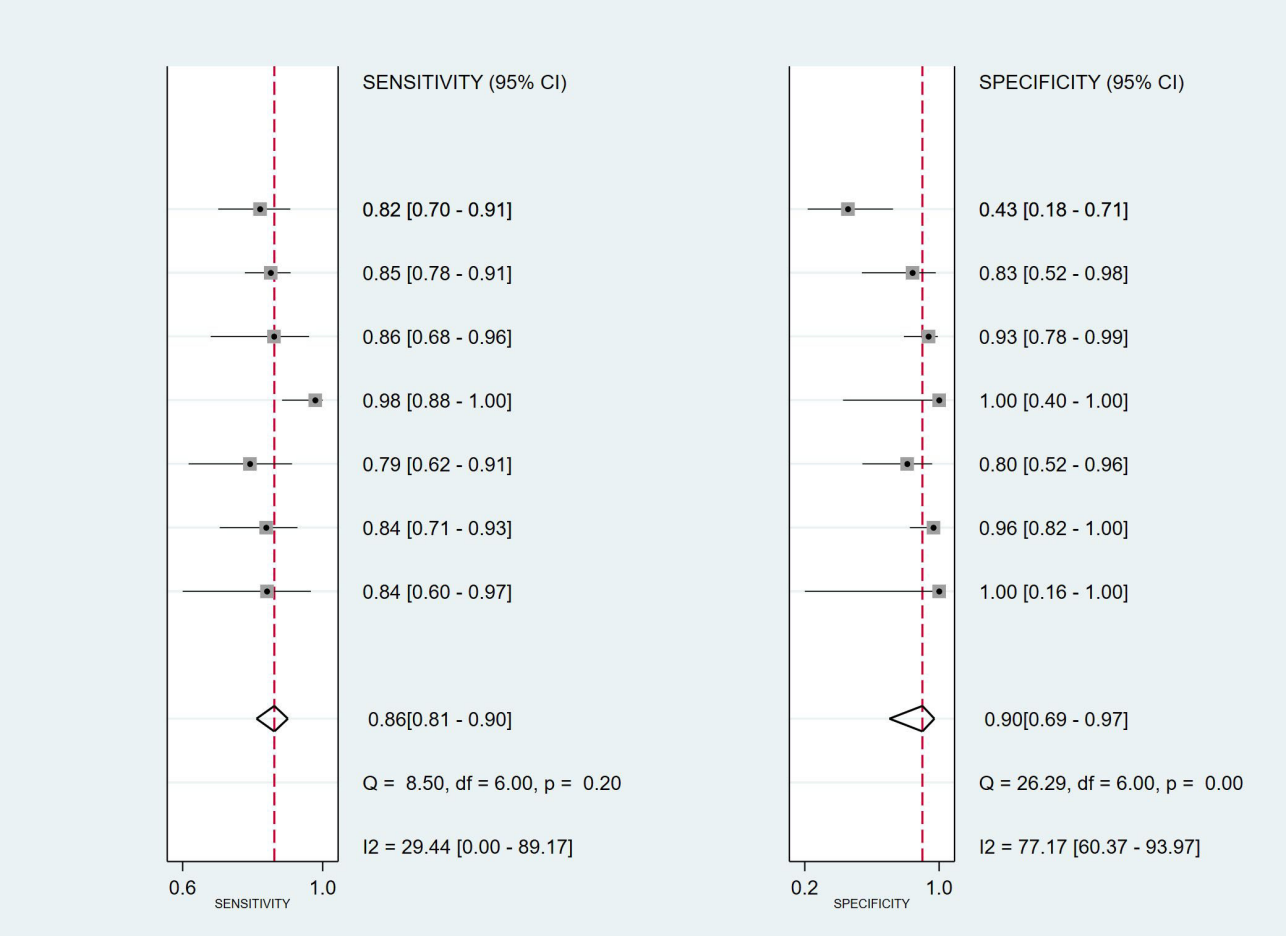
Combined forest plot for sensitivity and specificity, summarizing overall diagnostic performance.

**Figure 9:**
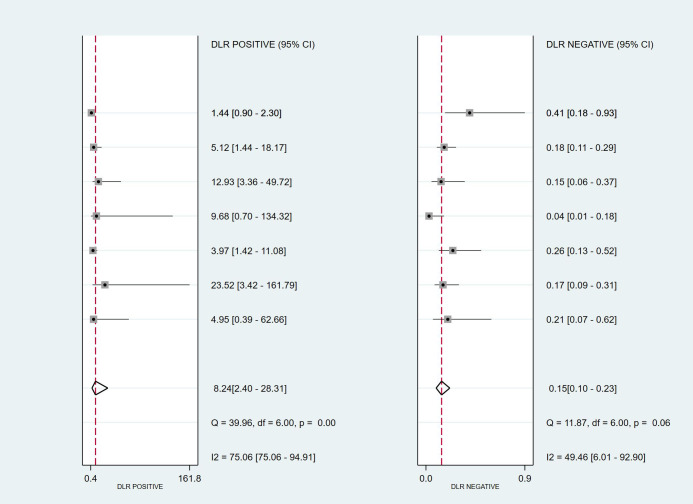
Forest plot for Likelihood Ratios (LR+ and LR-), combining the results of multiple studies.

**Figure 10:**
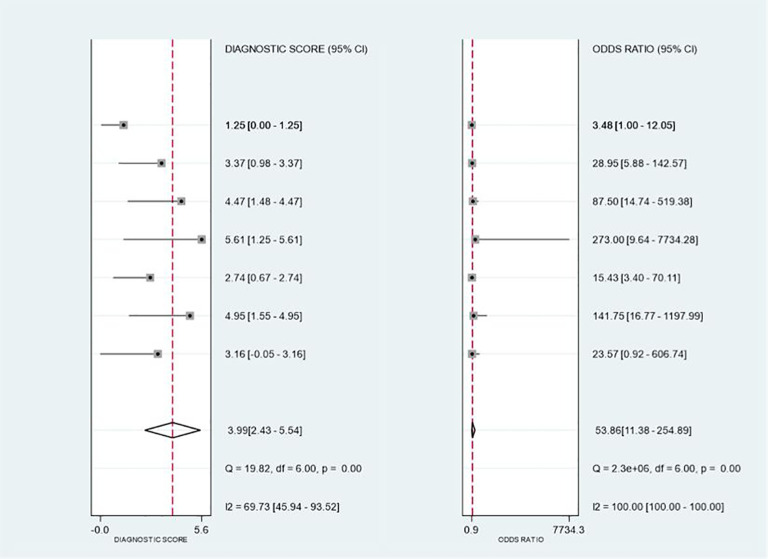
Forest plot for Diagnostic Odds Ratio (DOR) and Diagnostic Score, showing overall diagnostic accuracy.

As shown in **Figure 8**, mNGS demonstrated high sensitivity and specificity in diagnosing spinal infections. The pooled sensitivity was 0.86 (95% CI: 0.81–0.90), and specificity was 0.90 (95% CI: 0.69–0.97). **Figures 9**, **10** show a positive likelihood ratio (PLR) of 8.24 (95% CI: 2.40–28.31), a negative likelihood ratio (NLR) of 0.15 (95% CI: 0.10–0.23), and a diagnostic odds ratio (DOR) of 53.86 (95% CI: 11.38–254.89). The diagnostic score was 3.99 (95% CI: 2.43–5.54).

As shown in **Figure 9**, **10**, significant heterogeneity was observed among the studies. The Q-test and I² statistic indicated substantial heterogeneity (I² ≥ 50% or p < 0.1), suggesting that variability in study designs, sample types, or diagnostic methods may have contributed to the differences in results. However, due to the limited number of studies and the variations in diagnostic approaches, we did not perform further subgroup analyses to explore the sources of heterogeneity.

**Figure 11:**
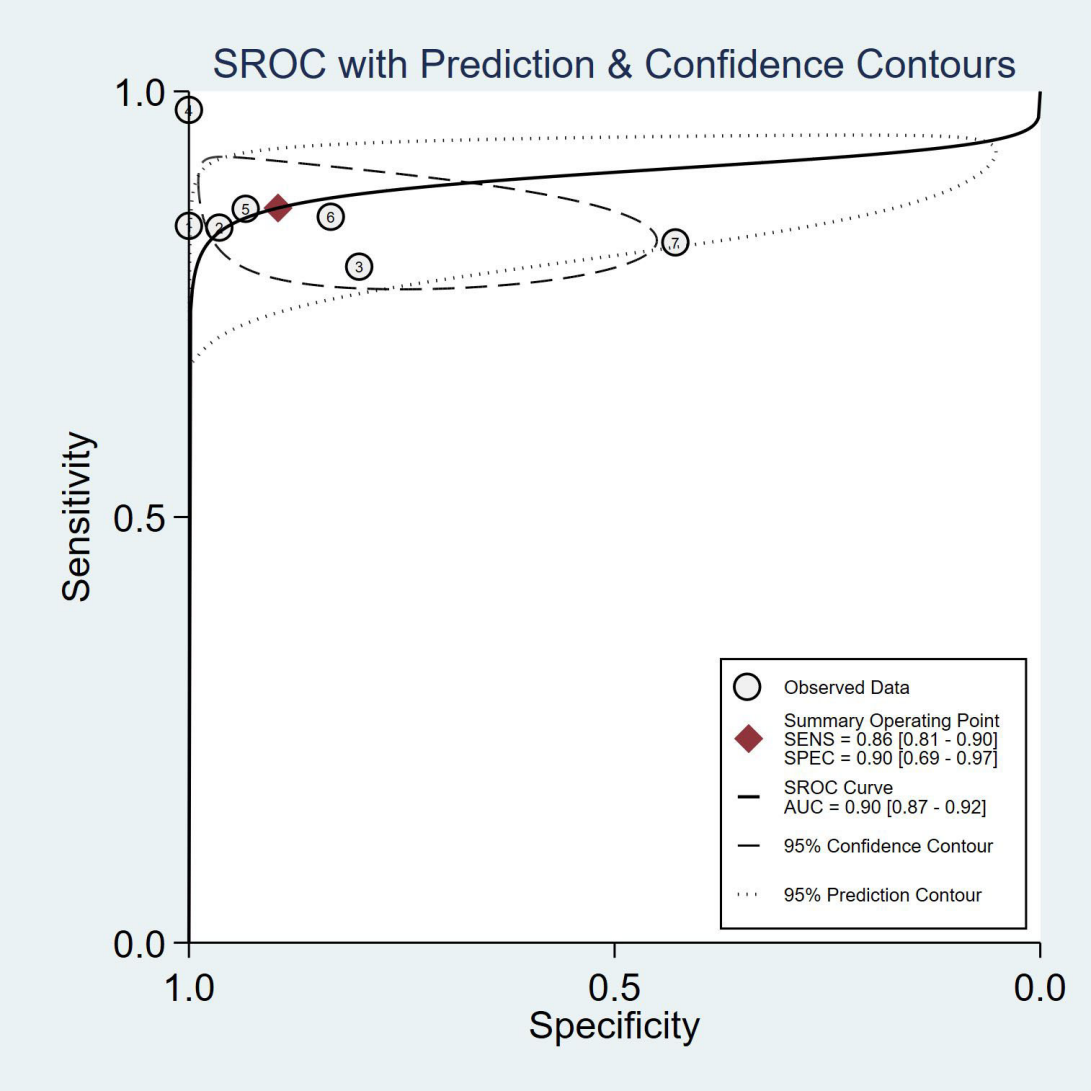
Summary Receiver Operating Characteristic (SROC) curve and the Area Under the Curve (AUC), indicating mNGS’s diagnostic performance.

The summary receiver operating characteristic (SROC) curve (**Figure 11**) indicated excellent diagnostic performance of mNGS, with an area under the curve (AUC) of 0.90 (95% CI: 0.87–0.92), suggesting high overall accuracy.

#### 3.5.2 Fagan nomogram analysis

**Figure 12:**
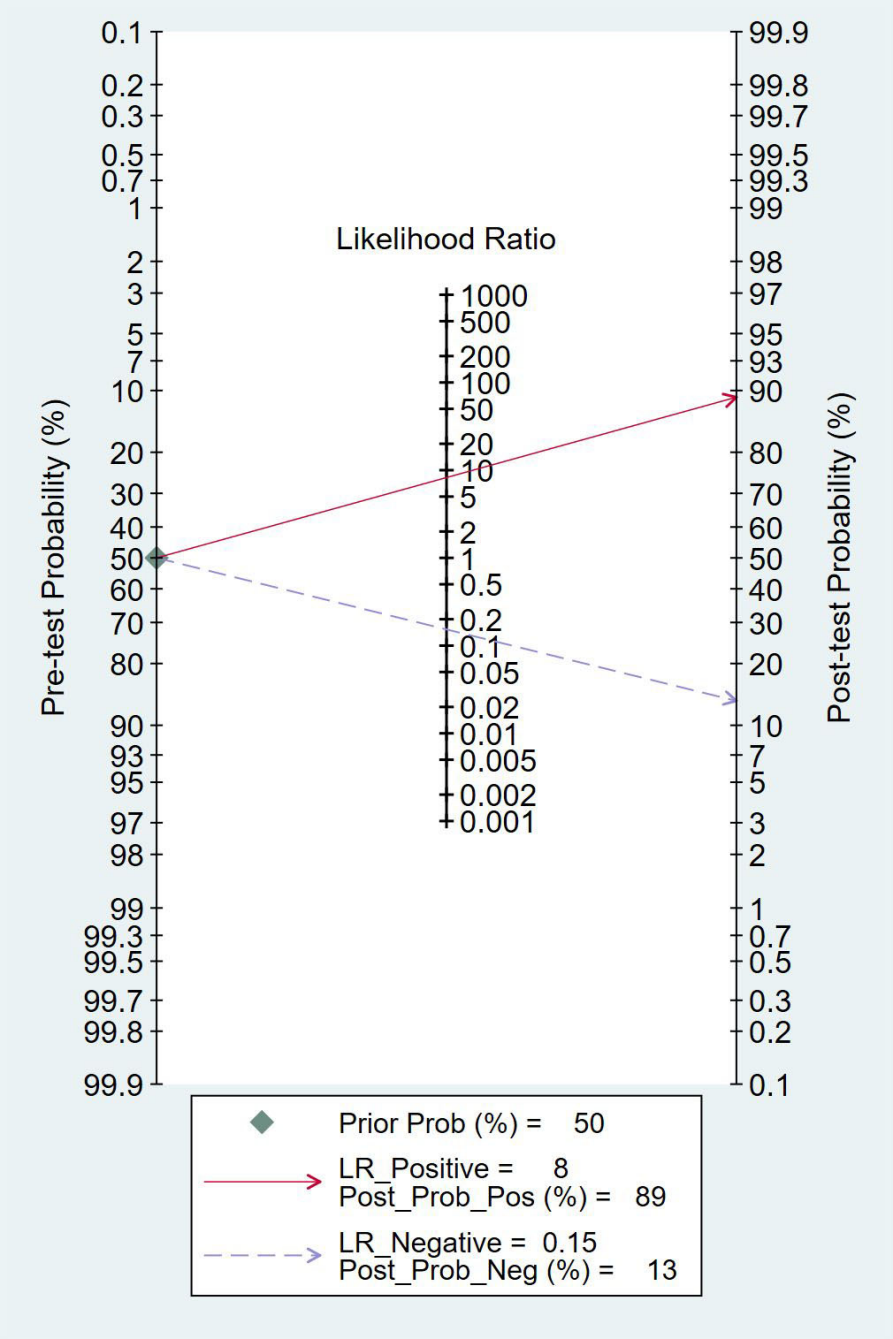
Fagan Nomogram illustrating the accuracy of mNGS in diagnosing spinal infection.

As shown in **Figure 12**, assuming a pre-test probability of 50%, a positive mNGS result increased the post-test probability to 89%, while a negative result reduced it to 13%. This further underscores the diagnostic utility of both positive and negative mNGS results.

#### 3.5.3 Publication bias

**Figure 13:**
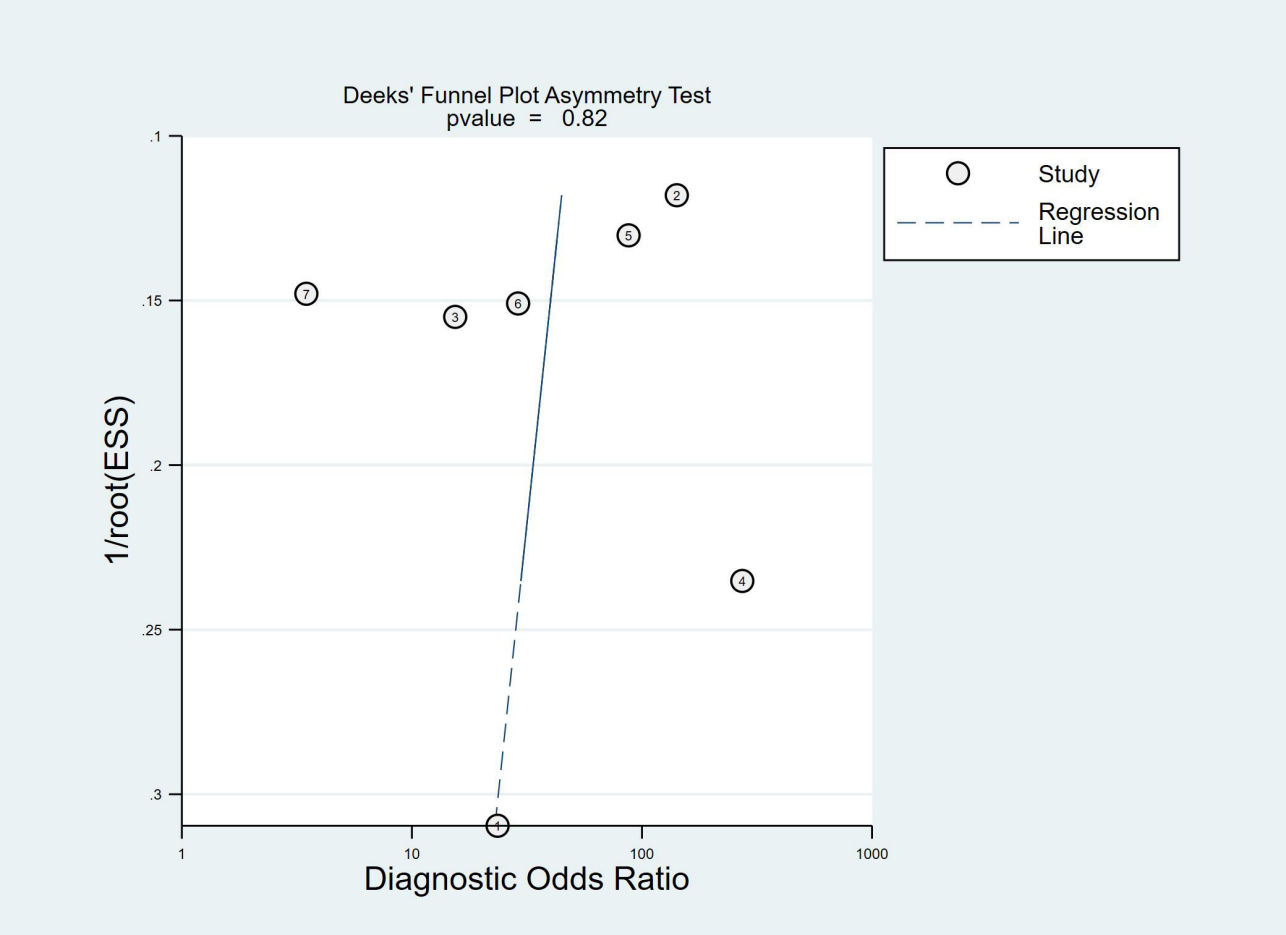
Deek’s Funnel Plot showing the assessment of publication bias across studies.

Deek’s funnel plot (**Figure 13**) yielded a slope coefficient of 0.82, indicating no significant publication bias among the included studies.

## 4 Discussion

Although mNGS has demonstrated excellent pathogen detection capabilities in a variety of infectious diseases—particularly in the diagnosis of challenging infections such as tuberculous meningitis and Chlamydia psittaci pneumonia (Tan et al., 2022)—its clinical application in the diagnosis of spinal infections remains controversial. At present, there is a lack of standardized testing procedures and universally accepted evidence, leaving the application of mNGS in this area still in an exploratory stage. Through a systematic literature search, we found that no systematic review or meta-analysis has yet been conducted to evaluate the diagnostic performance of mNGS in spinal infections. This evidence gap has to some extent restricted its standardized use in clinical practice and hindered clinicians’ objective understanding of its diagnostic utility. Therefore, this study aims to quantitatively assess the sensitivity, specificity, and overall diagnostic value of mNGS in spinal infections through a systematic review and meta-analysis, with the goal of providing a scientific basis for its broader adoption and standardization in clinical settings.

In our conventional meta-analysis, mNGS demonstrated significantly higher sensitivity and negative predictive value compared to traditional methods, suggesting its superior ability to detect true infections and rule out false negatives. While no significant differences were found in specificity and positive predictive value, this may be attributed to the limited number of included studies and small sample sizes in some analyses. Additionally, false-positive results due to contamination or colonization may also have influenced specificity estimates. Notably, mNGS achieved a pooled sensitivity of 86% and specificity of 90% in the diagnostic meta-analysis, with an area under the ROC curve of 0.90, indicating high overall diagnostic accuracy.

However, when interpreting the positive likelihood ratio (PLR) and negative likelihood ratio (NLR), the results of this study indicate that mNGS may not provide definitive diagnostic evidence by itself. In clinical diagnosis, a PLR > 10 or NLR < 0.1 is generally considered indicative of strong diagnostic performance. The PLR of 8.24 and NLR of 0.15 observed in our study do not meet these thresholds, suggesting that mNGS alone may not be sufficient to definitively diagnose or rule out spinal infections. Rather, mNGS should be viewed as a complementary diagnostic tool that may enhance the performance of traditional methods, particularly in challenging clinical cases.

Compared with conventional microbial culture, the principal advantage of metagenomic next-generation sequencing lies not in fully replacing culture, but in providing incremental pathogen detection and potentially faster reporting, thereby compensating for the limitations of culture in settings such as low pathogen burden, prior antibiotic exposure, or infections caused by fastidious organisms. In native pyogenic spinal infection, Li et al. directly compared the diagnostic performance of mNGS and culture and suggested that mNGS serves as a valuable adjunct to culture, improving etiologic yield and the completeness of clinical diagnosis (Li et al., 2025). Importantly, culture remains indispensable for antimicrobial susceptibility testing (AST) and standardized workflows; therefore, a more pragmatic clinical strategy is to incorporate mNGS into the routine microbiological workup and interpret it in conjunction with culture and, when indicated, histopathology. In line with this, Chen et al (Chen et al., 2024). proposed and evaluated a combined diagnostic approach integrating mNGS with microculture and pathology, emphasizing that sequencing results should be interpreted using integrated clinical, radiological, and pathological evidence to improve detection while reducing the risk of false-positive interpretation due to contamination or colonization. Beyond these complementary roles, mNGS enables broad-spectrum detection of bacteria, viruses, fungi, and parasites in a single assay without requiring prior assumptions about the causative pathogen, and in many cases offers a shorter turnaround time than culture-based methods—particularly for slow-growing or fastidious organisms. Accordingly, our review supports positioning mNGS as an adjunct to conventional culture, enhancing detection probability and potentially shortening turnaround time while preserving the critical role of culture in susceptibility-guided therapy.Nevertheless, several challenges remain in the clinical implementation of mNGS. First, its high cost and limited accessibility may prevent widespread use, particularly in resource-limited settings. Second, the interpretation of mNGS results requires integration of clinical, radiological, and laboratory data to differentiate between colonization, contamination, and true infection. Third, the lack of standardized procedures and reporting criteria across laboratories may result in inter-laboratory variability and limit the reproducibility of results. Finally, host background DNA and low pathogen abundance in some sterile site samples, such as vertebral biopsies, can further complicate analysis.

Nevertheless, several challenges remain in the clinical implementation of mNGS. First, its high cost and limited accessibility may prevent widespread use, particularly in resource-limited settings. Second, the interpretation of mNGS results requires integration of clinical, radiological, and laboratory data to differentiate between colonization, contamination, and true infection. Third, the lack of standardized procedures and reporting criteria across laboratories may result in inter-laboratory variability and limit the reproducibility of results. Finally, host background DNA and low pathogen abundance in some sterile site samples, such as vertebral biopsies, can further complicate analysis.

In terms of study quality, most of the included studies were retrospective and conducted in single centers in China, limiting generalizability. The lack of prospective multicenter studies and randomized controlled trials (RCTs) is a significant limitation of this analysis. Furthermore, the majority of studies had small sample sizes, with five studies including fewer than 50 cases, which may affect the robustness and generalizability of the findings. Heterogeneity in sample types, sequencing platforms, bioinformatics pipelines, and reference standards may also influence the pooled estimates. Although subgroup and sensitivity analyses were initially planned, the limited number of studies with comparable outcome measures prevented such analyses. As a result, the heterogeneity of the studies was assessed using the Q-test and I² statistic, and substantial heterogeneity (I² ≥ 50% or p < 0.1) was observed, which could have influenced the pooled results. Despite these limitations, our study represents the most comprehensive meta-analysis to date on the diagnostic performance of mNGS in spinal infections. The results provide valuable evidence supporting its clinical application, particularly as a complementary tool to conventional methods.

## 5 Conclusion

This systematic review and meta-analysis demonstrates that mNGS offers superior sensitivity and negative predictive value compared to traditional microbiological methods in the diagnosis of spinal infections. Additionally, the diagnostic meta-analysis confirmed its high overall accuracy, with excellent sensitivity, specificity, and area under the curve. These findings underscore the potential of mNGS as a valuable diagnostic tool, particularly in complex or atypical infections where conventional tests may fall short. This study provides significant evidence for the integration of mNGS into clinical practice, supporting its use as a complementary diagnostic approach alongside traditional methods. Future large-scale, prospective, and multicenter studies are essential to further validate these findings and to develop standardized protocols for its clinical implementation.

## Data Availability

The original contributions presented in the study are included in the article/**Supplementary Material**. Further inquiries can be directed to the corresponding author.
