# Supplementary material for: Comparative diagnostic performance of metagenomic next-generation sequencing and conventional microbial culture in spinal infections: a systematic review and meta-analysis
Source: Front Cell Infect Microbiol. 2026 Mar 13;16:1689254. doi: 10.3389/fcimb.2026.1689254 (PMC13021653; doi:10.3389/fcimb.2026.1689254)

**Appendix.**

**Supplementary data**

**Search Strategy;**

**PUBMED （55）**

((((spinal infections[Title/Abstract]) OR (spinal infection[Title/Abstract])) ) AND (((((((metagenomic sequencing[Title/Abstract]) OR (mNGS[Title/Abstract])) OR (metagenomic next-generation sequencing[Title/Abstract])) OR (shotgun metagenomics[Title/Abstract])) OR (genomics[Title/Abstract])) OR (genetic diagnosis[Title/Abstract])) OR (sequencing[Title/Abstract]))

**Cochrane Library （88）**

#1 spinal infections

#2 spinal infection

#3 #1 OR #2

#4 metagenomic sequencing

#5 mNGS

#6 shotgun metagenomics

#7 genomics

#8 genetic diagnosis

#9 sequencing

#10 #4 OR #5 #6 OR #7 OR #8 OR #9

#11 #3 AND #10

**Web of science （3077）**

1: metagenomic sequencing (Topic) OR mNGS (Topic) OR metagenomic next-generation sequencing (Topic) OR shotgun metagenomics (Topic) OR genomics (Topic) OR genetic diagnosis (Topic) OR sequencing (Topic)

2: spinal infections (Topic) OR spinal infection (Topic)

3: #2 AND #1 and Preprint Citation Index (Exclude – Database)

**Scopus （912）**

( ( TITLE-ABS-KEY ( metagenomic sequencing ) OR TITLE-ABS-KEY ( mNGS ) OR TITLE-ABS-KEY ( metagenomic next-generation sequencing ) OR TITLE-ABS-KEY ( shotgun metagenomics ) OR TITLE-ABS-KEY ( genomics ) OR TITLE-ABS-KEY ( genetic diagnosis ) OR TITLE-ABS-KEY ( sequencing ) ) ) AND ( ( TITLE-ABS-KEY ( spinal infections ) OR TITLE-ABS-KEY ( spinal infection ) ) )

**Supplementary Table 1: Full-text excluded studies with reasons for exclusion**

| **Author (Year)** | **Title** | **Country/Setting** | **Population/Condition** | **Index test(s)** | **Comparator(s)** | **Reference standard** | **Reason for exclusion** |
| --- | --- | --- | --- | --- | --- | --- | --- |
| Li (2023) | *Evaluation of different diagnostic methods for spinal tuberculosis infection* | China (Shandong; single center) | Suspected spinal TB (STB); 126 patients; 41 STB vs 85 non-STB | mNGS | MGIT-960 culture, T-SPOT.TB, Xpert MTB/RIF | Clinical diagnosis as reference (composite criteria) | Wrong target condition / not eligible population: study focuses on spinal tuberculosis and TB-specific diagnostic workflow (T-SPOT, Xpert, MGIT), not the target “spinal infection (pyogenic/non-TB) comparing mNGS vs conventional microbial culture” in our review. |
|  |  |  |  |  |  |  |  |
|  |  |  |  |  |  |  |  |
| Zhang (2024) | *Influence factors of metagenomic next-generation sequencing negative results in diagnosed patients with spinal infection* | China (Zhongshan Hospital, Fudan Univ., Shanghai; single center; Jan 2019–Feb 2023) | Suspected spinal infection 114; final infected 56 (per their gold standard) | mNGS (BGISEQ-50) | Routine culture (bacterial/fungal + MGIT960 for mycobacteria) | Either positive histopathology OR positive culture (or both) used as “gold standard” | Not eligible outcome/design: primarily investigates determinants of mNGS-negative results rather than providing extractable study-level data for our predefined comparison/meta-analysis framework. Also includes a substantial TB component, which does not match our target population if TB was excluded. |
| Li (2022) | *Diagnostic efficiency of metagenomic next-generation sequencing for suspected spinal tuberculosis in China: A multicenter prospective study* | China; 3 TB-specialized hospitals; prospective multicenter (Jan–Dec 2021) | Suspected spinal TB; final: spinal TB (n=38), non-TB spinal infection (n=53), non-infectious (n=9) | mNGS (MGISEQ-2000) | Conventional tests: aerobic/anaerobic bacterial culture, MGIT960, pathology, Xpert, T-SPOT.TB | Composite reference standard (CRS) with treatment response components for TB and non-TB infection classification | Wrong target condition / population mismatch: primary focus is suspected spinal tuberculosis in TB-specialized hospitals with TB-specific comparators (Xpert, T-SPOT, MGIT960) and mixed TB/non-TB case mix; not aligned with our review’s target if we restricted to non-TB/pyogenic spinal infections or to a uniform “mNGS vs conventional bacterial culture” framework. |
| Wan (2022) | *Potential clinical impact of metagenomic next-generation sequencing of plasma for cervical spine injury with sepsis in intensive care unit: A retrospective study* | China; ICU at Changzheng Hospital; Feb 2018–Jun 2019; retrospective | Cervical spine injury (CSI) with sepsis in ICU; 17 patients; 27 plasma samples | Plasma mNGS (cfDNA) (Illumina NextSeq550Dx) | Blood culture (and “combined culture” incl. sputum/urine/catheter drainage) | Culture used as gold standard in analyses (blood culture; or combined cultures) | Wrong target population/setting and index specimen: focuses on ICU sepsis in cervical spine injury using plasma cfDNA mNGS, not on spinal infection lesion specimens (tissue/pus/biopsy) and not the target diagnostic question (spinal infection pathogen detection by lesion-based mNGS vs conventional culture) in our review. |
| Li (2022) | *Case Report: Diagnosis of Primary Klebsiella pneumoniae in Cervical Spine by Metagenomic Next-Generation Sequencing* | China; Xiangya Hospital, Central South Univ. (single case) | Cervical suppurative infection due to Klebsiella pneumoniae (single patient) | Intraoperative specimen mNGS | Culture (intraoperative tissue culture; blood culture also reported) | Not a diagnostic accuracy study; narrative clinical confirmation (mNGS detected within ~20 h; culture confirmed later) | Study type not eligible: case report / single case, not an analytical diagnostic accuracy study and cannot provide extractable study-level 2×2 data for meta-analysis. |
| Du (2023) | *Case Report: Metagenomic Next-Generation Sequencing Confirmed a Case of Spine Infection with Brucella melitensis in Non-Endemic Area* | China; Second Affiliated Hospital of Zhejiang University (Hangzhou) | Single patient: 67-year-old man with spinal infection due to B. melitensis in non-endemic area | Biopsy tissue mNGS (CT-guided aspiration biopsy; mNGS on day 2) | “Traditional” culture reported as negative (blood/tissue cultures) | Not a diagnostic accuracy design; clinical synthesis (history + RBPT + imaging + response) | Study type not eligible: case report / single patient; not an analytical diagnostic accuracy study and cannot yield study-level 2×2 data for meta-analysis. |

**Supplementary Table 2:** Risk of bias summary for Retrospective cohort study: Reviewers' judgments about each risk of bias item per included Retrospective cohort study.

| Study | Selection | Comparability | Outcome |
| --- | --- | --- | --- |
| Zhang 2022 | ⭐⭐⭐⭐ | ⭐⭐ | ⭐⭐⭐ |
| Cheng 2023 | ⭐⭐⭐⭐⭐ | ⭐ | ⭐⭐⭐ |
| Wang 2023 | ⭐⭐⭐⭐⭐ | ⭐ | ⭐⭐⭐ |
| Zhang 2023 | ⭐⭐⭐⭐⭐ | ⭐⭐ | ⭐⭐⭐ |
| Xu 2022 | ⭐⭐⭐⭐ | ⭐⭐ | ⭐⭐⭐ |
| Ma 2022 | ⭐⭐⭐⭐⭐ | ⭐⭐ | ⭐⭐⭐ |
| Chen 2024 | ⭐⭐⭐⭐⭐ | ⭐⭐ | ⭐⭐⭐ |
| Lin 2023 | ⭐⭐⭐⭐⭐ | ⭐⭐ | ⭐⭐⭐ |
| Wang 2024 | ⭐⭐⭐⭐ | ⭐⭐ | ⭐⭐⭐ |
| Yin 2025 | ⭐⭐⭐⭐⭐ | ⭐ | ⭐⭐⭐ |
| Li 2025 | ⭐⭐⭐⭐ | ⭐⭐ | ⭐⭐⭐ |
| Qi 2024 | ⭐⭐⭐⭐⭐ | ⭐ | ⭐⭐⭐ |
| Huang 2023 | ⭐⭐⭐⭐ | ⭐⭐ | ⭐⭐⭐ |
| Lv 2024 | ⭐⭐⭐⭐⭐ | ⭐ | ⭐⭐⭐ |

**Supplementary Figure 1:**

1. The funnel plots of publication bias in Positive Agreement.


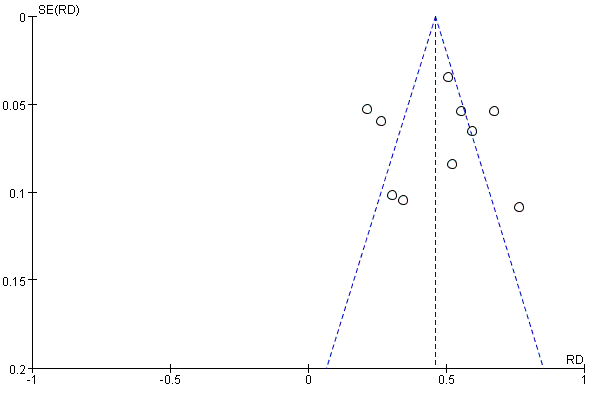


1. The funnel plots of publication bias in Sensitivity.


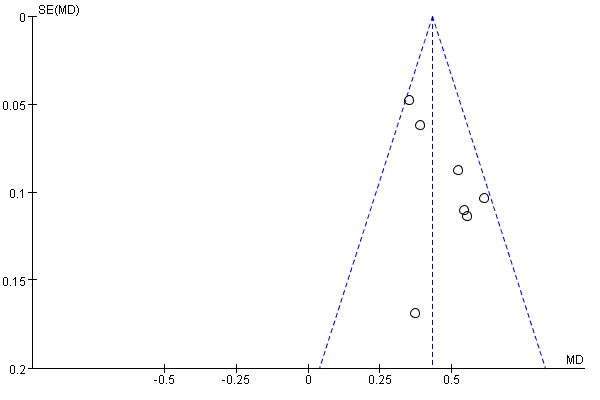


1. The funnel plots of publication bias in Specificity.


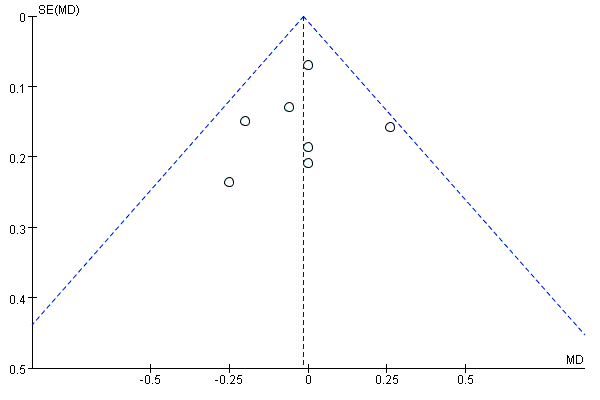


1. The funnel plots of publication bias in PPV.


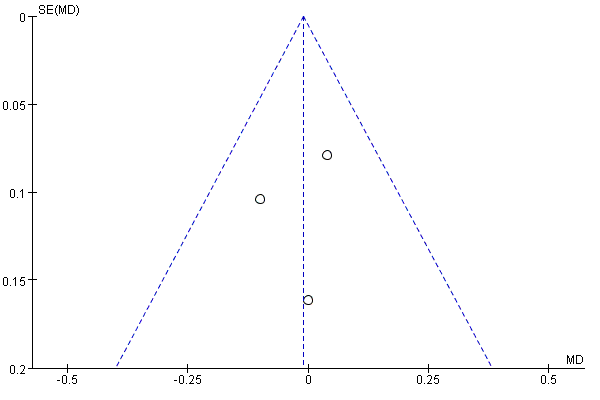


1. The funnel plots of publication bias in NPV.


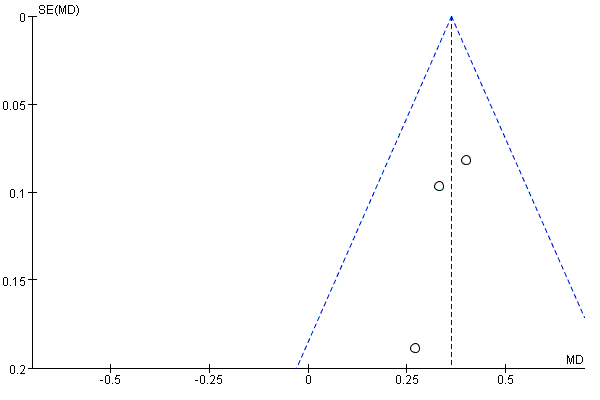

Supplement: Supplementary file 1 [file DataSheet1.docx]
